# Gene Expression of Abcc2 and Its Regulation by Chicken Xenobiotic Receptor

**DOI:** 10.3390/toxics12010055

**Published:** 2024-01-10

**Authors:** Yanhong Gao, Huacheng Deng, Yuying Zhao, Mei Li, Liping Wang, Yujuan Zhang

**Affiliations:** 1 Jiangsu Key Laboratory of Sericultural Biology and Biotechnology, School of Biotechnology, Jiangsu University of Science and Technology, Zhenjiang 212100, China; 17852408215@163.com (Y.G.); deng.hcvet@foxmail.com (H.D.); leeknowcatleebit@163.com (Y.Z.); ch3cooha@163.com (M.L.); 2 College of Veterinary Medicine, Nanjing Agricultural University, Nanjing 210095, China; wlp71@163.com

**Keywords:** Abcc2, CXR, induction, chicken

## Abstract

Membrane transporter multidrug resistance-associated protein 2 (MRP2/Abcc2) exhibits high pharmaco-toxicological relevance because it exports multiple cytotoxic compounds from cells. However, no detailed information about the gene expression and regulation of MRP2 in chickens is yet available. Here, we sought to investigate the expression distribution of Abcc2 in different tissues of chicken and then determine whether Abcc2 expression is induced by chicken xenobiotic receptor (CXR). The bioinformatics analyses showed that MRP2 transporters have three transmembrane structural domains (MSDs) and two highly conserved nucleotide structural domains (NBDs), and a close evolutionary relationship with turkeys. Tissue distribution analysis indicated that Abcc2 was highly expressed in the liver, kidney, duodenum, and jejunum. When exposed to metyrapone (an agonist of CXR) and ketoconazole (an antagonist of CXR), Abcc2 expression was upregulated and downregulated correspondingly. We further confirmed that Abcc2 gene regulation is dependent on CXR, by overexpressing and interfering with CXR, respectively. We also demonstrated the induction of Abcc2 expression and the activity of ivermectin, with CXR being a likely mediator. Animal experiments demonstrated that metyrapone and ivermectin induced Abcc2 in the liver, kidney, and duodenum of chickens. Together, our study identified the gene expression of Abcc2 and its regulation by CXR in chickens, which may provide novel targets for the reasonable usage of veterinary drugs.

## 1. Introduction

Multidrug resistance-associated protein 2 (MRP2, encoded by Abcc2 gene) is a member of the ATP-binding cassette (ABC) superfamily of transmembrane proteins [1]. The human Abcc2 gene was first cloned from cisplatin-resistant human cancer cell lines, where it was shown to limit drug accumulation. One year later, it was cloned from human fibroblasts. The human Abcc2 gene has 32 exons and is located on chromosome 10q24 [2]. The intron exon structure of the Abcc2 gene has a high degree of similarity to other members of the ABC family, such as Abcb1 [3,4,5]. Subsequently, Abcc2 gene clones were generated from many species. MRP2 utilizes the energy obtained from ATP hydrolysis to actively efflux out various substrates from cells [6]. Importantly, a large variety of structurally unrelated drugs (e.g., doxorubicin, vincristine, daunorubicin, grepafloxacin, and saquinavir) [7,8] and endogenous compounds (e.g., conjugated steroid hormones, bile salts, and leukotriene C4) are actively transported by MRP2 [9,10,11,12]. MRP2 is highly expressed in pharmacologically important organs such as the intestine, liver, and kidneys of humans and rodents [13,14,15], and therefore, it plays an important role in the pharmacokinetics and toxicity of substrate drugs [16]. Thus, understanding the mechanisms involved in MRP2 expression and function is of great importance.

There is evolving evidence for the role of nuclear receptors in the induction of MRP2. Pregnane X receptor (PXR) and constitutive androstane receptor (CAR) that are activated by structurally diverse compounds (e.g., therapeutic drugs and environmental toxicants) are crucial nuclear receptors in the induction of MRP2 [17,18,19]. Both nuclear receptors are characterized by the presence of a typical, well-conserved DNA-binding domain and a variable substrate-binding domain [20]. PXR and CAR play important roles in the pharmacokinetics of a broad spectrum of endogenous and xenobiotic compounds by regulating the transcriptional expression of cytochrome P450s and ABC transporters [21,22]. For example, research has shown that PXR and CAR agonists play major roles in abrogating metabolic pathophysiology (e.g., cholestasis and hypercholesterolemia) [23,24,25]. However, PXR is also an important mediator of adverse metabolic phenotypes (e.g., enhances acetaminophen’s toxicity) and drug–drug interactions (e.g., enhances drug resistance) [26]. Therefore, identifying important molecules and understanding their molecular mechanisms involved in MRP2 regulation is fundamentally relevant for understanding the pharmacokinetics and toxicity of substrate drugs.

Chicken xenobiotic receptor (CXR) has high homology with PXR and CAR, and it was proven to regulate the xenobiotic-metabolizing enzymes CYP2H1 and CYP2C45 [27]. CXR is highly expressed in drug metabolism-related tissues, such as the liver, kidneys, and small intestine [27]. Our previous studies showed that CXR agonists affect the pharmacokinetic behavior of ABC substrate drugs by regulating the expression of the ABC protein. However, the regulatory mechanism of chicken MRP2 is not fully understood.

This study aimed to investigate the gene expression of Abcc2 and explore whether CXR regulates the expression and activity of MRP2. Here, we incorporated a comprehensive bioinformatics analysis of chicken MRP2 and examined Abcc2 mRNA expression in various tissues of chicken. Furthermore, we determined that CXR directly regulates the expression of chicken MRP2, and that ivermectin could induce chicken Abcc2 expression and function through activated CXR. These studies suggest that ligands for CXR activate the transcription of the MRP2 gene, which may cause drug–drug interactions or promote the excretion of toxic agents out of cells.

## 2. Materials and Methods

### 2.1. Materials and Cell Culture

Ivermectin, metyrapone, and ketoconazole were purchased from Sigma Aldrich Chimie (St. Quentin Fallavier, France). 2′,7′-Dichlorodihydrofluorescein diacetate (CDFDA) was purchased from MedChemExpressed LLC’ (Shanghai). DNA and RNA transfection reagents were purchased from Yisheng Biotechnology (Shanghai) Co., Ltd. PrimeScript™ RT reagent Kit with gDNA Eraser (Perfect Real Time) and Trizo were purchased from Takara Biomedical Technology (Beijing) Co., Ltd. A 2× Taq Master Mix was purchased from Vazyme Biotech Co., Ltd. Dulbecco’s Modified Eagle’s Medium (DMEM) was purchased from Thermo Fisher Scientific. Trypsin–EDTA solution was purchased from Solarbio Science & Technology Co., Ltd.

Chicken primary hepatocytes were isolated from the livers of 14-day-old chicken embryos, as described previously [28]. In brief, the liver was extracted from embryonic eggs under sterile conditions. After sterile cutting into small pieces, we incubated the liver tissue in a trypsin solution at 37 °C for 10–15 min. We collected liver cells by centrifugation (1000 rpm, 5 min) and filtered them through a 150 µm sieve. The obtained liver cells were cultured in serum-free M199 medium supplemented with 100 IU/mL penicillin, 100 µg/mL streptomycin, 5 µg/mL transferrin, 2 mM glutamine, and 1 µg/mL hydrocortisone, and maintained at 37 °C, 5% CO_2_, and 95% humidity in a cell incubator.

### 2.2. Bioinformatics Analysis and Protein Structural Models of Chicken MRP2

A sequence analysis of chicken MRP2 was performed using NCBI (http://www.ncbi.nlm.nih.gov/, accessed on 12 October 2023) BLAST. Multiple alignments of MRP2 protein sequences were generated using the BioEdit program (https://bioedit.software.informer.com/7.2, accessed on 10 October 2023). Phylogenetic analysis was used to determine the affinities of chicken MRP2 with those of the other animals, and the alignments were imported into MEGA version 7 (https://www.megasoftware.net/, accessed on 8 October 2023) to generate a phylogenetic tree as the first structures of MRP2. The secondary structures of chicken and human MRP2 were predicted using Protter1.0 software (http://wlab.ethz.ch/protter/start/, accessed on 8 October 2023) and Protter1.0 server for 3D structures [29].

### 2.3. Molecular Modeling

Molecular docking was used to verify the binding activity between active ingredients (metyrapone, ketoconazole, and ivermectin) and key targets (CXR). AutoDock Vina (Vina, version 1.1.2) was used as the molecular docking program in this study. The SWISSMODEL server was used to model the protein sequence. The structures of small molecules of metyrapone, ketoconazole, and ivermectin were downloaded from the Pubchem database. The protein was designated as the receptor, and the compound was designated as the ligand. PyMOL (4.3.0 version) software (https://pymol.org/, accessed on 27 October 2023) was used to remove organic matter, and AutodockTools (http://mgltools.scripps.edu/downloads, accessed on 27 October 2023) was used to hydrogenate, check the charge, specify the atomic type as AD4 type, and construct the docking grid box of the protein structure. In addition, the chemical composition (small-molecule ligand) should determine the Root and select the reversible bond of the ligand in AutodockTools. Finally, in AutodockTools, both the protein structure and the small-molecule-ligand format were converted from “.PDB“ to “.PDBQT“ for further docking. After docking with Vina, the scores of protein and small molecule docking combinations were calculated, and the force analysis and visualization of three-dimensional and two-dimensional angles were performed using Discovery Studio software 2019.

### 2.4. Animals and Tissue Sample Collection

The tissue samples (heart, liver, spleen, lung, kidney, duodenum, jejunum, ileum, colorectal, cecum, and stomach) of 30-week-old chickens (6 chickens per group) were collected, and RNA was extracted to analyze the mRNA expression of Abcc2. Tissue samples of all chickens were collected, quickly frozen in liquid nitrogen, and stored at −70 °C for further analysis. The treatment of chicken tissue was implemented in accordance with the guidelines of the Jiangsu Provincial Science and Technology Commission (approval No. 2022-0007) and Nanjing Agricultural University.

### 2.5. RNA Isolation, RT-PCR, and PCR Analysis

The design of mRNA primers corresponding to chicken MRP2 was based on the previous literature, and they were sent to Zhejiang Shangya Biological Company for synthesis [30]. The tissue samples of different experimental chickens were collected. After grinding the tissues, the RNA of the corresponding tissues was extracted using the Trizol method. RNAs were quantified using a photometer (Eppendorf, Hamburg, Germany) at 260/280 nm and ranged from 1.8 to 2.0. The integrities of each RNA sample were verified with electrophoresis on a 1.4% agarose gel and adjusted to a uniform concentration. Then, the RNA was reverse-transcribed into cDNA using a reverse transcription kit. The Abcc2 mRNA was detected using RT-PCR with β-actin as an internal reference. The RT-PCR detection system consisted of upstream and downstream primers, cDNA, and RNase-free water. The cDNA of different tissues of chickens was used as a template, and the mRNA primers of MRP2 were used as upstream and downstream primers. The 2× Taq Master Mix and RNase-free water were added to make up a 25 μL ordinary PCR system. The PCR products were electrophoresed with 1% agarose gel, and the results were observed on the imager after electrophoresis in WD-9413B Gel Imaging Analysis System (LiuYi Biotechnology, Beijing, China). The primer sequences are listed in Table 1.

### 2.6. Flow Cytometry Analysis

The primary hepatocytes of 14-day-old chicken embryos were isolated and collected. After being cultured in serum-free DMEM medium for 24 h, the cells of the experimental groups were treated with ivermectin (5 and 10 μM), ketoconazole (30 μM), and metyrapone (500 μM), respectively. After 24 h of drug stimulation, 10 μM CDFDA (a specific fluorescent substrate of MRP2) was added and incubated for 30 min [31]. The cells were digested into a single-cell suspension using trypsin, and they were washed 3 times with PBS. The fluorescence of CDFDA was detected using flow cytometry.

### 2.7. SiRNA and Overexpression of CXR

When chicken primary hepatocytes were cultured to about 70% confluence in 12-well plates, the CXR overexpression plasmid named pcDNA3.1-CXR was transfected, and the cells were treated with 500 µM metyrapone for 24 h. Subsequently, the cells were collected for the analysis of Abcc2 expression. Similarly, primary chicken liver cells were cultured to 70% fusion and transfected with CXR-targeting siRNA (siCXR) or a negative control, disrupting siRNA (NC siRNA) for 24 h. Then, the cells were treated with 500 µM metyrapone for 24 h and harvested for the analysis of ABCC2 expression. The overexpression plasmids and interfering sequences were previously constructed in the laboratory [32].

### 2.8. Statistical Analysis

In this study, all results are expressed as the mean ± S.D. Statistical analysis was conducted using SPSS software (version 20.0, SPSS Inc., Chicago, IL) with one-way ANOVA and post hoc Tukey’s test. *p* < 0.05 represents a significant difference, while *p* < 0.01 represents a highly significant difference.

## 3. Results

### 3.1. Sequence Analysis of Chicken MRP2

**Figure 1:**
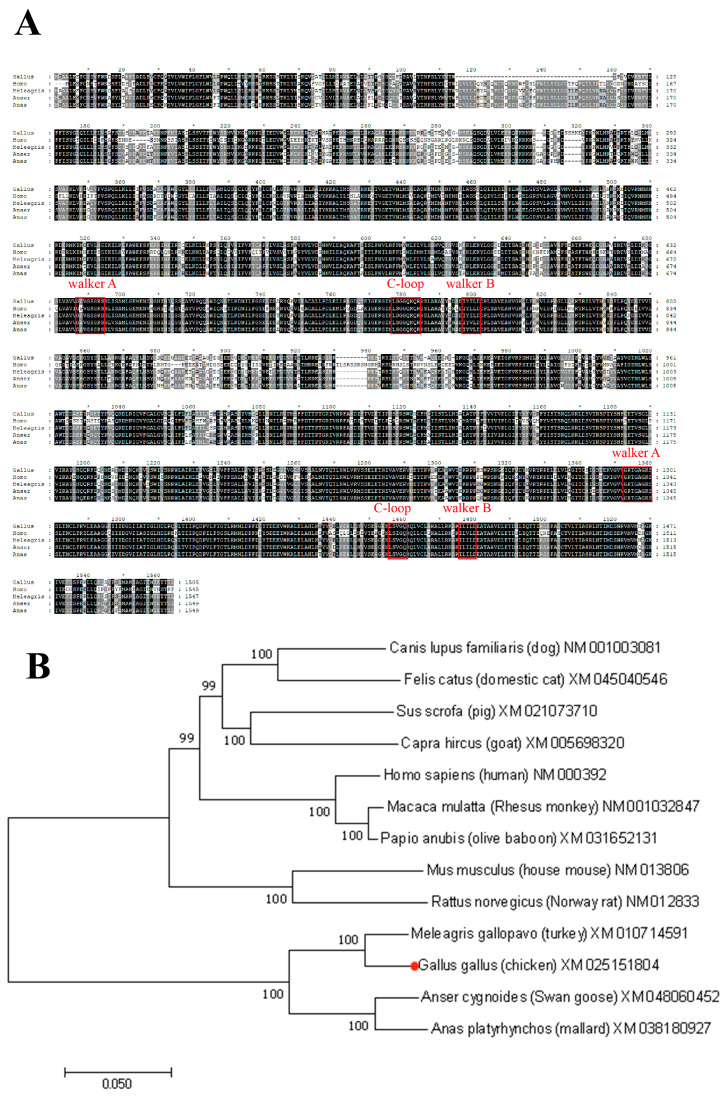
Bioinformatics analysis of chicken Abcc2. (**A**) Multiple-sequence alignments of amino acid sequence of chicken Abcc2 with other species with MEGA 7.0. GenBank IDs: Gallus gallus, XM_025151804; Homo sapiens, NM_000392; Anser cygnoides, XM_048060452; and Anas platyrhynchos (mallard), XM_038180927. There are 10 amino acids between each * and the following numbers, and 20 amino acids between two *. (**B**) Phylogenetic tree of Abcc2 coding sequences. Calculations were performed using the ClustalW algorithm, and evolutionary histories were inferred using the neighbor-joining method in MEGA 7.0.

We used the online website InterPro to analyze the chicken Abcc2 sequence. The results showed that the conserved amino acid sequences of the ABC protein family Walker A (GXXGXGKST) and Walker B (hhhhD) were present in the chicken Abcc2 sequence. In addition, the second feature of the ABC family, C-loop, also exists in the chicken Abcc2 sequence, and it has 15 residues and usually starts with LSGGQ (Figure 1A). The results of the phylogenetic tree and the sequence alignment showed that chicken and turkey belong to the same branch, and the homology of MRP2 sequence is highly similar to that of turkey (XM_010714591), goose (XM_048060452), and duck (XM_038180927), at 92.2%, 85.3%, and 84.6%, respectively. Results indicate that the above Abcc2 samples are homologous. However, the chicken and mammal branches are far away; the former’s homology with the human MRP2 sequence is 59.9% (Figure 1B).

**Figure 2:**
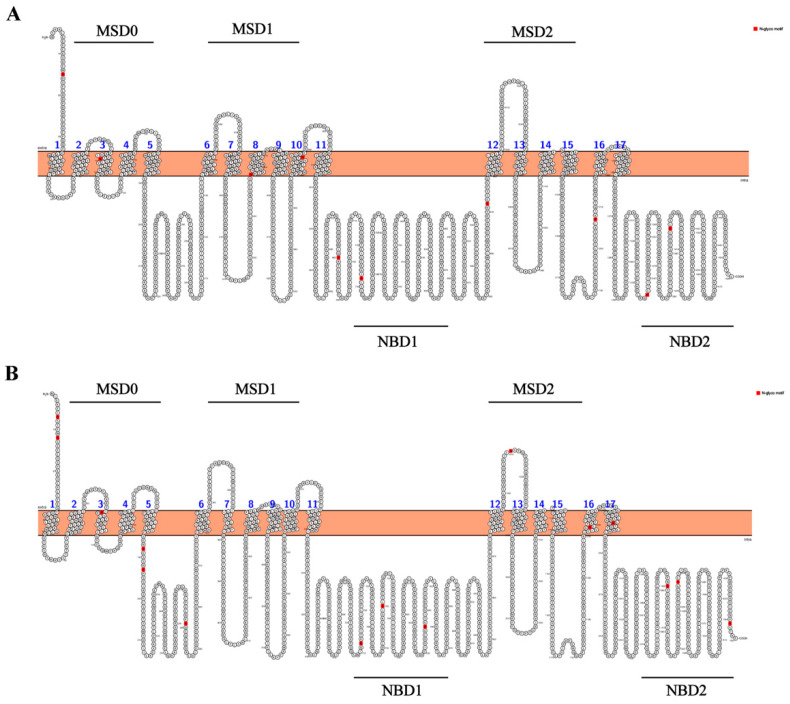
Secondary structure prediction of (**A**) chicken MRP2 and (**B**) human MRP2. The potential N-glycosylation sites for both chicken and human MRP2 are highlighted in red, indicating 10 sites in chicken and 15 sites in human.

**Figure 3:**
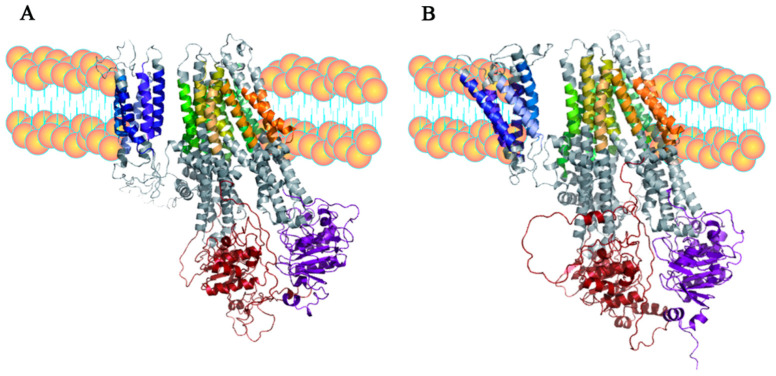
The 3D structure of MRP2: (**A**) chicken MRP2 and (**B**) human MRP2. Blue color indicates MSD0, green color indicates MSD1, yellow color indicates MSD2, red color indicates NBD1, and purple color indicates NBD2.

The secondary structure model of chicken MRP2 protein was derived from DeepTMHMM (https://dtu.biolib.com/DeepTMHMM, accessed on 8 October 2023). The secondary structure of chicken and human MRP2 was exported using Protter 1.0 software (http://wlab.ethz.ch/protter/start/, accessed on 8 October 2023). Chicken MRP2 is predicted to have 17 transmembrane helices, with 3 transmembrane structural domains (MSDs) and 2 highly conserved nucleotide structural domains (NBDs), like human MRP2 (Figure 2A,B). The tertiary structure of chicken MRP2 contains 44 α-Spirals and 18 β-folds. However, the tertiary structure of human MRP2 contains 48α-Spirals and 23 β-folds (Figure 3A,B).

### 3.2. Abcc2 mRNA Expression in Chicken Tissues

**Figure 4:**
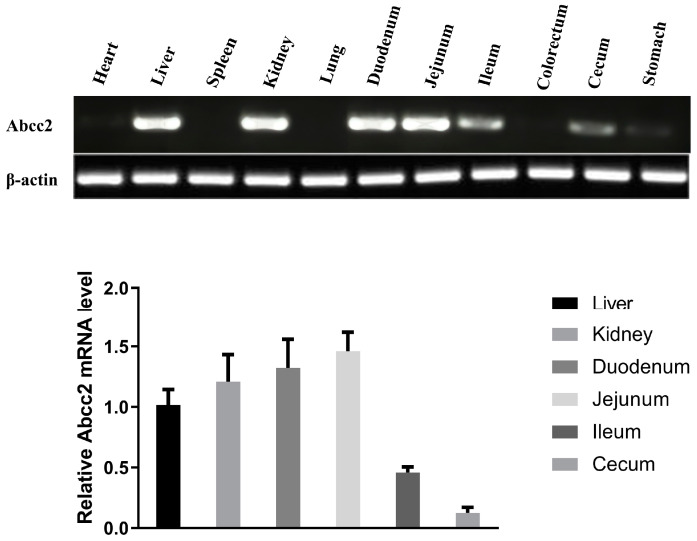
MRP2 expression in different tissues of chicken. (**A**) PCR and (**B**) RT-PCR were used to analyze the expression of MRP2 mRNA in different tissues of chickens. Bars show means ± SD of at least six independent experiments.

The expression levels of chicken MRP2 mRNA varied in different tissues of the chicken. The expression profile of MRP2 in chicken tissues shows that MRP2 is mainly expressed in the liver, kidney, duodenum, and jejunum, with low expression in the ileum and cecum. However, MRP2 mRNA expression was not detected in the tissues of the heart, spleen, lung, colorectum, and stomach (Figure 4A,B).

### 3.3. Docking of CXR with Small Molecules

**Figure 5:**
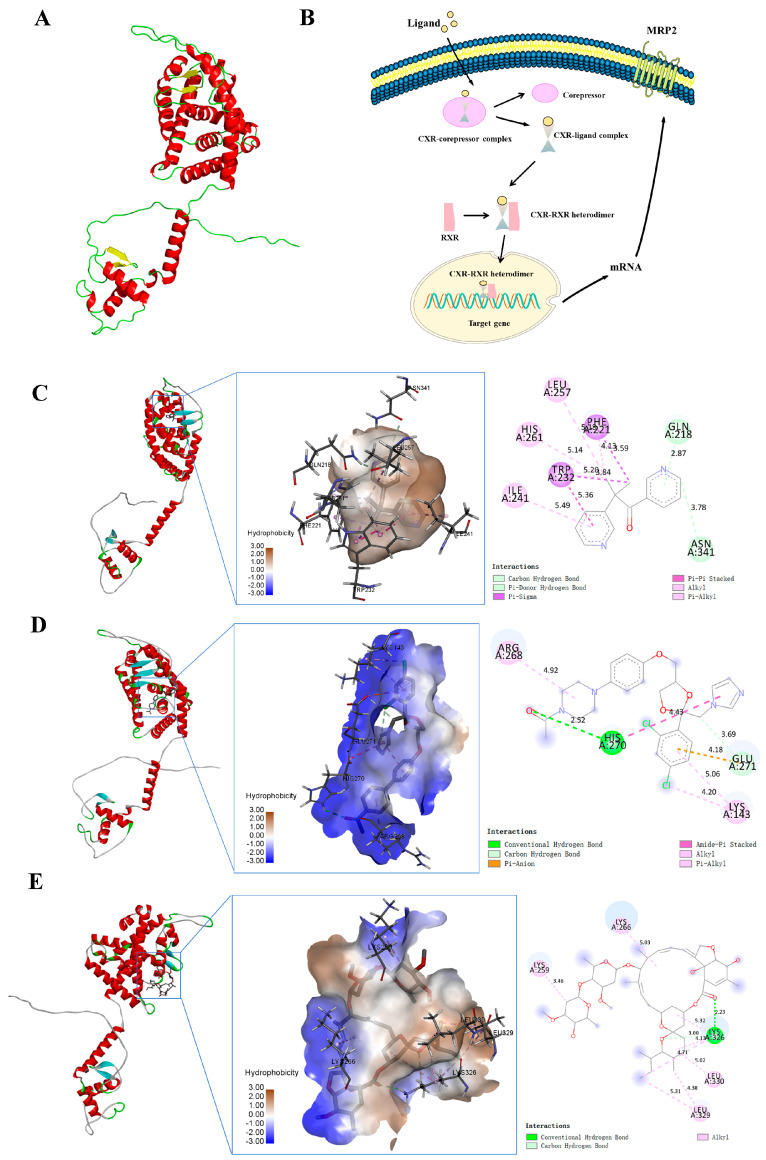
Docking of CXR with small molecules and analysis of its regulation mechanism on MRP2. (**A**) CXR tertiary structure. (**B**) Regulation mechanism of MRP2 by nuclear receptor CXR. (**C**) Molecular docking of CXR with metyrapone. (**D**) Molecular docking of CXR with ketoconazole. (**E**) Molecular docking of CXR with ivermectin. In the two-dimensional diagram, pink, purple, and brown dashed lines all represent hydrophobic forces; in the three-dimensional diagram, the green dashed line represents hydrogen bonds, and the light green dashed line represents carbon hydrogen bonds.

The tertiary structure of CXR was predicted with the SWISS-MODEL online server, and the tertiary structure of CXR contained 17α-helices and 4 β-strands (Figure 5A). CXR is an exogenous ligand-dependent nuclear receptor. After the exogenous compound ligand enters the cell, it can separate the nuclear receptor CXR from the corepressor protein to form a CXR ligand complex. This complex then combines with RXR to form a CXR-RXR heterodimer, which is then transferred into the nucleus and bound to the corresponding binding site, thereby promoting the transcription and translation of target genes. When the ligand is a CXR activator, it promotes the above pathways, while CXR antagonists inhibit the signaling pathway (Figure 5B). The molecular docking results showed that the binding energies between MRP2 protein and small molecules such as metyrapone, ketoconazole, and ivermectin were −8.4 kcal/mol, −6.6 kcal/mol, and −7.5 kcal/mol, respectively. If the binding energy between the ligand and the target protein is less than −5, then the ligand and receptor protein can stably bind. Therefore, these small molecules can stably bind to the protein CXR. From the three-dimensional diagram, it can be seen that the ligand small-molecule metyrapone can bind to the 232 TRP amino acid residue of the receptor-modeling protein through three hydrophobic forces of 5.36 Å, 3.84 Å, and 5.20 Å, respectively (Figure 5C). The ligand small-molecule ketoconazole can bind to the 270 HIS amino acid residue of the receptor-modeling protein through two hydrogen bonds of 2.52 Å and a hydrophobic force of 4.43 Å, respectively (Figure 5D). The ligand small-molecule ivermectin can bind to the 329 LEU amino acid residue of the receptor-modeling protein through two hydrophobic forces of 5.31 Å and 4.38 Å, respectively (Figure 5E). Due to the presence of these forces, the above small molecules can stably bind to receptor protein CXR. We further validated the interaction between CXR and these small molecules through experiments.

### 3.4. CXR Dependence of Abcc2 Induction

**Figure 6:**
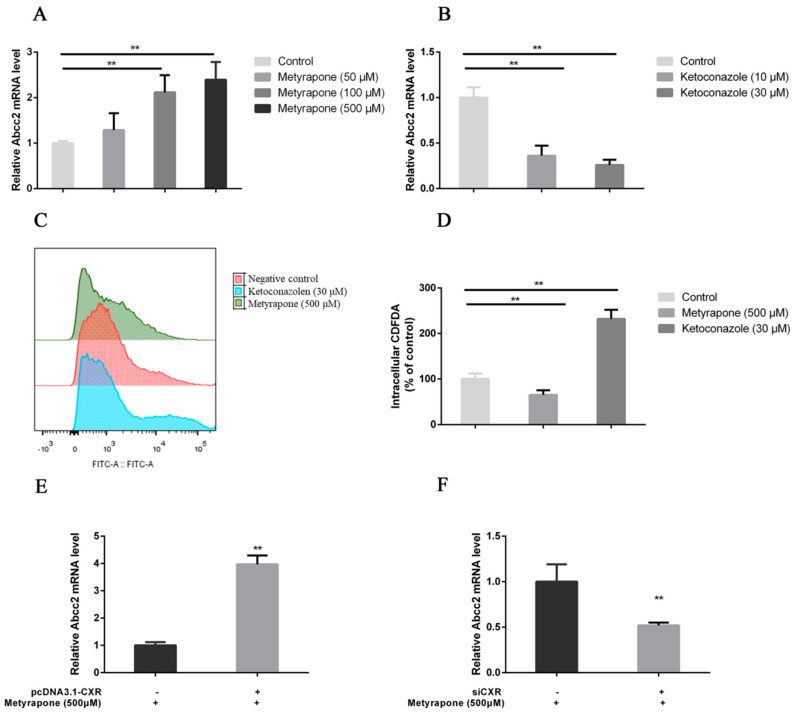
CXR dependence of Abcc2 induction. (**A**) Expression of MRP2 by the CXR agonist metyrapone in chicken embryo primary hepatocytes. (**B**) Expression of MRP2 by the CXR antagonist ketoconazole in primary hepatocytes of chicken embryos. (**C**,**D**) Representative results and summary of CDFDA accumulation. Histograms show fluorescence (*x*-axis), indicating CDFDA accumulation, as a function of cell number (*y*-axis). (**E**) RT-PCR was used to analyze the mRNA expression of Abcc2 in chicken primary hepatocytes treated with 500 μM metyrapone for 24 h after transfection with CXR expression vector. (**F**) Expression of Abcc2 mRNA in chicken primary hepatocytes treated with 500 μM metyrapone 24 h after siRNA-mediated CXR knockdown. Bars show means ± SD of at least three independent experiments. ** *p* < 0.01.

To verify the regulatory effect of CXR on Abcc2 expression, we first tested whether the CXR activator metyrapone and CXR antagonist ketoconazole regulate the expression of Abcc2 in chicken primary hepatocytes. The results showed that metyrapone with a concentration of 50, 100, and 500 μM significantly upregulated the expression of Abcc2 by 1.29-, 2.12-, and 2.40-fold compared to that of the control group, respectively (Figure 6A). However, ketoconazole with a concentration of 10 and 30 μM significantly downregulated the expression of Abcc2 by 0.36- and 0.26-fold compared to that of the control group, respectively (Figure 6B). To determine whether the induction of Abcc2 expression by CXR modulators regulates the transport function of MRP2, a CDFDA (a selective MRP2 substrate) accumulation experiment was conducted in chicken primary hepatocytes. The results showed that intracellular CDFDA fluorescence was 60% lower in cells pretreated with metyrapone than in untreated cells (*p* < 0.01), while 232% higher in cells pretreated with ketoconazole (Figure 6C,D). These results indicate that the CXR-induced expression of Abcc2 is related to the MRP2 transport function. Through the implementation of overexpressing and RNA interference experiments, we further validated whether the direct participation of CXR in the induction of Abcc2 expression is caused by metyrapone. The results showed that when CXR was overexpressed, the expression of Abcc2 was 3.97 times higher than that of the control group, and the expression of Abcc2 was significantly increased (*p* < 0.01) (Figure 6E). When CXR was inhibited, the expression of Abcc2 was significantly reduced by 0.52 times compared to that of the Abcc2 control group after treatment with metyrapone (*p* < 0.01) (Figure 6F). In addition, the results shown in the Supplementary Materials show that siCXR significantly decreased CXR mRNA expression by 79%, compared with cells that were transfected with the NC siRNA. Overall, these data indicate that CXR is directly involved in the regulation of Abcc2.

### 3.5. Ivermectin-Activated CXR Induces Abcc2 Expression and Function

**Figure 7:**
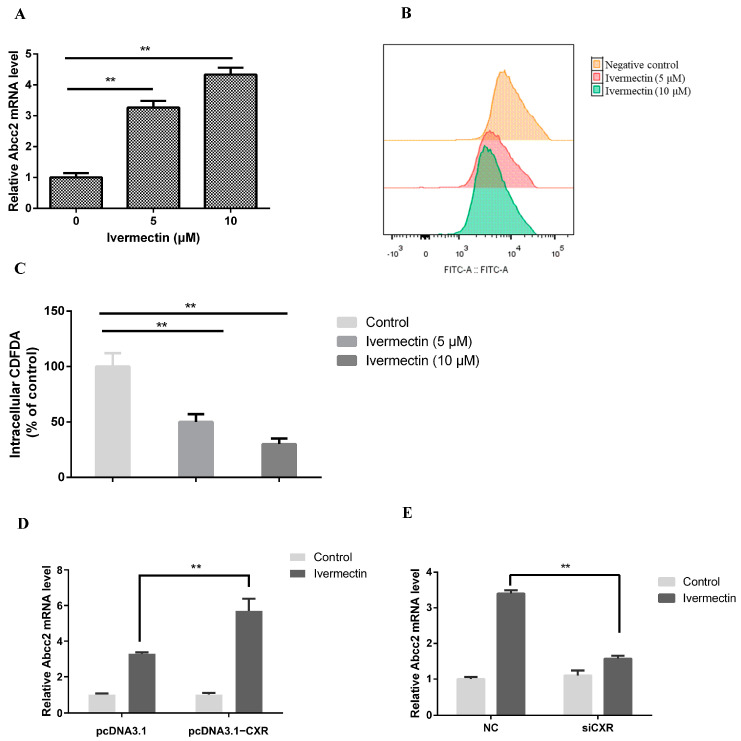
Ivermectin regulates Abcc2 expression through activation of CXR. (**A**) RT-PCR assay for MRP2 expression after treatment of chicken embryo primary hepatocytes with ivermectin (5 μM, 10 μM). (**B**,**C**) Representative results and summary of CDFDA accumulation. (**D**) Detection of Abcc2 expression in chicken embryo primary hepatocytes transfected with a CXR expression vector or (**E**) siRNA and then treated with 10 μM ivermectin. Bars show means ± SD of at least three independent experiments. ** *p* < 0.01.

Our previous studies have shown that ivermectin can activate CXR. Here, we further investigated whether ivermectin induces the expression and function of MRP2 through the CXR pathway. The results showed that ivermectin with concentration of 5 and 10 μM significantly upregulated the expression of Abcc2 2.27- and 4.34-fold compared to the control group, respectively (Figure 7A). Accordingly, intracellular CDFDA fluorescence was 50% and 30% lower in cells pretreated with 5 and 10 μM ivermectin than in untreated cells (*p* < 0.01) (Figure 7B,C). Further experiments showed that Abcc2 overexpression in chicken primary hepatocytes significantly enhanced ivermectin-induced Abcc2 mRNA expression (3.3 vs. 5.7-fold, *p* < 0.01) (Figure 7D). In contrast, using siCXR to knock down CXR weakened the induction of Abcc2 by ivermectin (3.4- vs. 1.57-fold, *p* < 0.01) (Figure 7E). These data indicate that ivermectin-activated CXR induces Abcc2 expression and function in chicken primary hepatocytes.

### 3.6. CXR-Mediated Induction of Abcc2 in Chicken

**Figure 8:**
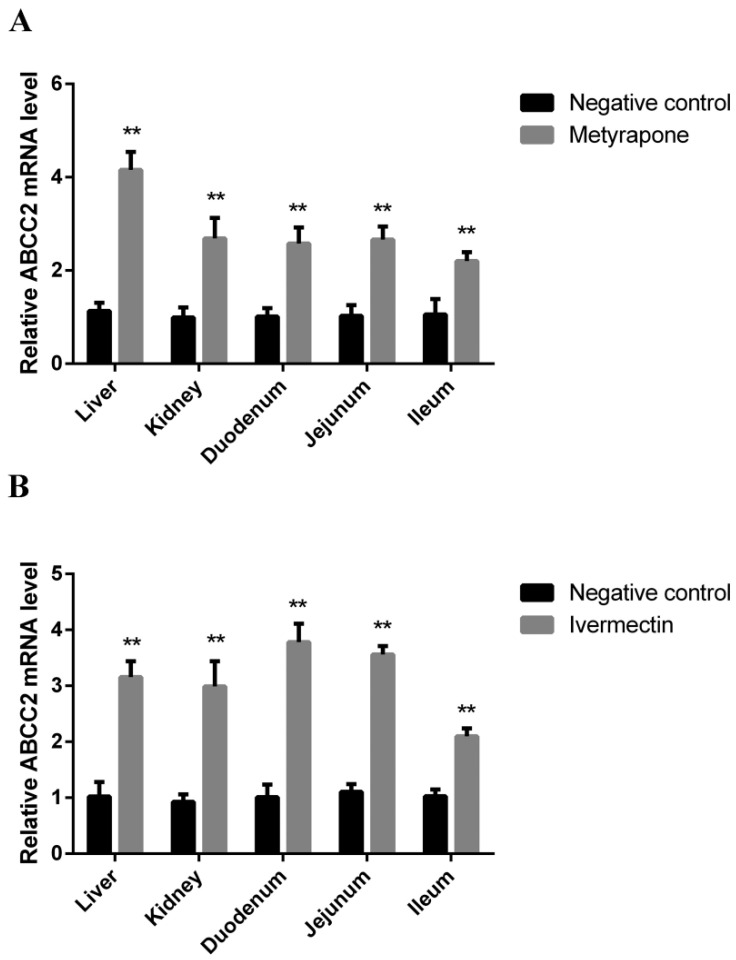
Expression of Abcc2 in different tissues of CXR activator metyrapone- and ivermectin-treated chickens. RT-PCR analysis of the expression levels of Abcc2 mRNA in liver, kidney, duodenum, jejunum, and ileum for metyrapone (**A**) and ivermectin (**B**). Bars show means ± SD of at least three independent experiments. ** *p* < 0.01.

We further validated whether CXR regulates the expression of Abcc2 through in vivo experiments. The results showed that after CXR agonist metyrapone treatment, the expression levels of Abcc2 in chicken liver, kidney, duodenum, jejunum, and ileum tissues were 4.16, 2.69, 2.58, 2.66, and 2.2 times higher than those in the control group (*p* < 0.01) (Figure 8A). Similarly, after ivermectin treatment, the expression levels of Abcc2 in the liver, kidney, duodenum, jejunum, and ileum were 3.16, 2.99, 3.78, 2.56, and 2.10 times that of the control group (*p* < 0.01) (Figure 8B).

## 4. Discussion

MRP2 was formerly known as the canalicular multispecific organic anion transporter, and it is involved in the transport of organic anions, including bile salts, as well as glutathione [33]. Natural mutations in the MRP2 gene are the molecular basis of Dubin–Johnson syndrome/hyperbilirubinemia II, a disorder associated with conjugated hyperbilirubinemia in humans [34,35,36]. In addition, the nuclear receptor-mediated induction of MRP2 has been reported to affect the pharmacokinetics and toxicity of substrate drugs [16]. However, the gene expression and regulation of MRP2 in chicken is poorly understood. In this study, we explored the expression distribution of Abcc2 and the regulatory effects of CXR on the expression of chicken MRP2.

The integrated bioinformatical analysis could improve our understanding of the underlying molecular mechanism of how MRP2 works on the barrier tissues [37,38]. The chicken MRP2 shared high similarities with MRP2 from turkey and Anas platyrhynchos, indicating that their physiological functions are similar [39]. The presence of the conserved WalkerA and WalkerB in each NBD is a unique feature in these transporters [40,41]. MRP2 is highly expressed in the liver, kidneys, and small intestine of chickens, which are important pharmacological sites that affect substrate drug absorption and metabolism, as well as participate in the excretion of toxic compounds from the body. Notably, MRP2 is active in the transport of xenobiotics such as the anticancer drugs cisplatin and vinca alkaloids [42,43]. In addition to MRP2, several other transporter proteins, including P-gp and BCRP, are localized in pharmacologically important organs [37,44]. In fact, the clinical drug interactions induced by the combination of ABC substrates and inhibitors or inducers may be partly due to the presence of transport proteins in pharmacologically important tissues [45,46]. These findings provide additional connections between various transporter proteins and the toxic excretion of various compounds.

Clarifying the molecular mechanism regulating MRP2 expression has important clinical significance. Metyrapone induces MRP2 mRNA expression in chicken. Due to the proven ability of metyrapone to activate CXR, the MRP2 gene appears to be a target for activated CXR [27]. However, it is uncertain whether other nuclear receptor pathways are involved in the regulatory effect of metyrapone on MRP2. To rule out this possibility, the experiment further used RNA interference and overexpression techniques to manipulate the CXR state of cells. Results showed that the induction of BCRP by metyrapone was significantly enhanced and weakened due to the overexpression and silencing of CXR, confirming that CXR directly regulates the expression of BCRP [32]. Consistent with the in vitro results, animal experiments showed that metyrapone significantly induces the expression of MRP2 in the liver, kidneys, duodenum, and jejunum of chickens. In conclusion, the present study showed a novel role of CXR in the regulation of MRP2. Since MRP2 is localized in pharmacologically important sites and functions to transport a variety of compounds out of cells, our data suggest that the activation of CXR is likely to enhance the excretion of these compounds from the body. CXR also mediates the transcriptional induction of hepatic CYP2H1, which, in turn, metabolizes many endogenous and exogenous compounds. This coordinate regulation results in a net clearance of compounds from the liver and an efflux of these compounds into the intestine and from the body [33]. Overall, this study further expands the role of CXR, from the regulation of exogenous metabolic enzymes to the regulation of the ABC transporter family. In addition, we found that ivermectin, a macrocyclic lactone widely used in the animal treatment of helminth infections and external parasites, can induce the expression and function of chicken MRP2 through the CXR signaling pathway. This result suggests that the drug–drug interaction caused by the long-term induction of MRP2 by drugs should be considered in clinical practice.

## 5. Conclusions

In summary, this study modeled and analyzed the structure of the MRP2 protein and explored its mRNA expression levels in different tissues from chickens. Further, we validated that CXR is a regulatory factor for the expression of MRP2. Therefore, exogenous substances may alter the pharmacokinetic characteristics of MRP2 substrate drugs through nuclear receptor-mediated pathways. Our results explain the possible molecular mechanisms of drug–drug interactions and provide the possibility of improving drug treatment outcomes by improving the efficacy and reducing the toxicity of MRP2 substrate drugs.

## Figures and Tables

**Table 1:** Oligonucleotide sequences of primers.

Name	Primer Sequence (5′–3′)
Abcc2 (MRP2)-F	CTGCAGCAAAATGAGAGGACAATG
Abcc2 (MRP2)-R	CAGAAGCGCAGAGAAGAAGACCAC
β-actin-F	TGCGTGACATCAAGGAGAAG
β-actin-R	TGCCAGGGTACATTGTGGTA

## Data Availability

The data presented in this study are available upon request from the corresponding authors.

## Supplementary Materials

The following supporting information can be downloaded at: https://www.mdpi.com/article/10.3390/toxics12010055/s1, Figure S1: Knockdown of CXR.
