# Supplementary material for: Gene Expression of Abcc2 and Its Regulation by Chicken Xenobiotic Receptor
Source: Toxics. 2024 Jan 10;12(1):55. doi: 10.3390/toxics12010055 (PMC10818656; doi:10.3390/toxics12010055)
Supplement: Supplementary file 1 [file toxics-12-00055-s001.zip › toxics-2757740-supplementary.pdf]

Supplementary Material

Article title： Gene Expression of Abcc2 and Its Regulation by Chicken Xenobiotic Receptor

Journal name： Toxics

Author names： Yanhong Gao, Huacheng Deng, Yuying Zhao, Mei Li, Liping Wang and Yujuan Zhang

***Corresponding author:** Yujuan Zhang (zhangyujuan907@163.com)

Jiangsu Key Laboratory of Sericultural Biology and Biotechnology, School of Biotechnology, Jiangsu University of Science and Technology, Zhenjiang 212100, China.

Figure S1

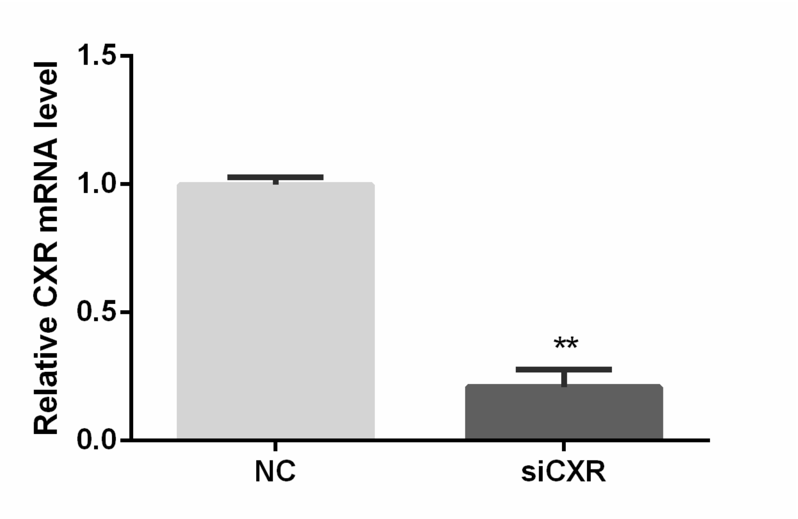

Figure S1. Knockdown of CXR. Knockdown of CXR mRNA in chicken primary hepatocytes after 12 h of transfection with negative control (NC)-scrambled siRNA or chicken CXR-specific siRNA (siCXR). Data are represented as mean ± SD (N = 4). Bars show means ± SD of at least three independent experiments. ** $P < 0.01$.
